# PKC-Mediated ZYG1 Phosphorylation Induces Fusion of Myoblasts as well as of *Dictyostelium* Cells

**DOI:** 10.1155/2012/657423

**Published:** 2012-02-06

**Authors:** Aiko Amagai, Harry MacWilliams, Takahiro Isono, Mariko Omatsu-Kanbe, Shinya Urano, Kazuo Yamamoto, Yasuo Maeda

**Affiliations:** ^1^Graduate School of Life Sciences, Tohoku University, Sendai 980-8577, Japan; ^2^Zoologisch Institute, Ludwig-Maximilians Universitat, 80333 München, Germany; ^3^Central Research Laboratory, Shiga University of Medical Science, Otsu, Shiga 520-2192, Japan; ^4^Department of Physiology, Shiga University of Medical Science, Otsu, Shiga 520-2192, Japan

## Abstract

We have previously demonstrated that a novel protein ZYG1 induces sexual cell fusion (zygote formation) of *Dictyostelium* cells. In the process of cell fusion, involvements of signal transduction pathways via Ca^2+^ and PKC (protein kinase C) have been suggested because zygote formation is greatly enhanced by PKC activators. In fact, there are several deduced sites phosphorylated by PKC in ZYG1 protein. Thereupon, we designed the present work to examine whether or not ZYG1 is actually phosphorylated by PKC and localized at the regions of cell-cell contacts where cell fusion occurs. These were ascertained, suggesting that ZYG1 might be the target protein for PKC. A humanized version of *zyg1* cDNA (*mzyg1*) was introduced into myoblasts to know if ZYG1 is also effective in cell fusion of myoblasts. Quite interestingly, enforced expression of ZYG1 in myoblasts was found to induce markedly their cell fusion, thus strongly suggesting the existence of a common signaling pathway for cell fusion beyond the difference of species.

## 1. Introduction

Some species of *Dictyostelium* exhibit dimorphism in development: sorocarp formation as an asexual development and macrocyst formation as a sexual development. In the asexual development, amoeboid cells grow and multiply feeding on bacteria during the vegetative growth phase. Upon exhaustion of external nutrients, starving cells stop growing and enter the differentiation phase. They gather together to form cell aggregates. A tip is formed on the top of each cell aggregate, which then migrates as a slug-shaped mass. After migration, the slug changes its shape dramatically to form a sorocarp consisting of a stalk with an apical mass of spores. 

In the sexual development, *Dictyostelium mucoroides 7 *(Dm7), one species of cellular slime molds, forms macrocysts homothallically without its opposite mating type. Macrocyst formation in Dm7 is characterized by the formation of large aggregates after starvation, which are then subdivided into smaller masses (precysts), each of which is surrounded by a fibrillar sheath. At the center of each precyst, there arises a cytophagic cell (a giant cell), which in turn engulfs all the other cells in the precyst. The engulfed cells (endocytes) are eventually broken down into granular remnants. The enlarged cytophagic cell finally becomes surrounded by a thick wall to form a mature macrocyst [1]. After a resting period, the macrocyst germinates to release several amoeboid cells and initiates a new life cycle [2]. Other species of cellular slime molds, such as *Dictyostelium discoideum* (*D. discoideum*), form macrocysts heterothallically with their opposite mating types [3, 4].

 The giant cell which appears during macrocyst formation is clarified as a zygote which is formed by cell fusion and subsequent nuclear fusion [5]. Several molecules have been identified as regulators of zygote formation. Ethylene has been known as a potent inducer, while cAMP as an inhibitor [5, 6]. Ca^2+^ is also able to induce zygote formation because the efficiency of zygote formation is significantly elevated by the presence of extracellular Ca^2+^ [6–8]. Ca^2+^ is supposed to activate PKC (protein kinase C) in the signal transduction pathways involved in zygote formation. In this connection, phorbol esters such as TPA (12-*O*-tetradecanoyl phorbol-13-acetate), potent activators of PKC, have been shown to enhance zygote formation [9, 10]. In contrast, staurosporine, an inhibitor of kinases including PKC, inhibits zygote formation markedly [9, 10] and therefore macrocyst formation [11]. It is quite possible that zygotes in heterothallic strains may be formed by essentially the same mechanism as that in homothallic strains [10, 12].

 In the process of myoblast fusion, cells cease dividing and change their morphology from fibroblast-like to spindle (bipolar) shape. End-to-end alignment of cells is formed along with lateral-to-lateral alignment in a process termed recognition-alignment. Alignment is followed by adhesion, defined as the stage immediately prior to membrane union. The highly elongated cells of typical myoblast fusion, in which nuclei were arranged in a single file, were finally formed. Many molecules which regulate myoblast fusion have been reported [13, 14]. Among them, it has been well documented that the signaling pathway mediated by Ca^2+^ and PKC is closely involved in cell fusion of myoblasts during myogenesis [15–19]. Since the signaling pathway via Ca^2+^ and PKC is involved in myoblast fusion as well as in *Dictyostelium* cell fusion, we have interested to know if there is a functionally ZYG1-like protein capable of inducing cell fusion in myoblasts. 

 As a gene involved in sexual fusion (zygote formation) during *Dictyostelium* development, *zyg1 *cDNA was isolated from *Dm7* by differential screening [20]. The *zyg1* gene encodes a novel protein (ZYG1; deduced Mr 29.4 × 10^3^) consisting of 268 amino acids. It was predicted that it has neither transmembrane domains nor specified signal sequences although ZYG1 protein has predicted PKC-mediated phosphorylation sites. The expression of *zyg1 *begins after 2 h of starvation and reaches its maximum level at 8 h under submerged culture conditions. The *zyg1* expression pattern is quite similar to the temporal change of zygote formation during sexual development (marcocyst formation) [11]. In the transformants overexpressing *zyg1, *the formation of zygotic giant cells is greatly enhanced, and macrocysts are formed even under unfavorable conditions for macrocyst formation in wild type Dm7 cells [20]. Interestingly, the *zyg1* expression is induced by ethylene, a potent plant hormone [21]. 

 In general, the activated PKC is known to translocate to the cell membrane in oocytes [22]. Thus, it is possible that ZYG1 may translocate to the cell membrane where cell fusion occurs and is phosphorylated by PKC because ZYG1 is a likely substrate for PKC.

 The present work was undertaken to answer the following questions. (1) Where is ZYG1 protein localized in *Dictyostelium* cells? (2) Is ZYG1 protein actually phosphorylated by PKC? (3) Can ZYG1 protein induce cell fusion in myoblasts as well as in *Dictyostelium *cells? The results obtained have showed that ZYG1 protein phosphorylated by PKC is localized at the region where cell fusion occurs and that ZYG1 protein also induces cell fusion in myoblasts.

## 2. Materials and Methods

### 2.1. Cell Culture and Developmental Conditions

*Dictyostelium discoideum* Ax-2, its transformants (GFP^CONT^ and GFP/ZYG1^OE^), and mouse myoblasts (C2C12) were used in this work. Vegetative cells of Ax-2 were grown axenically in PS-medium (1% Special Peptone (Oxoid: Lot no. 333 56412), 0.7% Yeast extract (Oxoid), 1.5% D-glucose, 0.11% KH_2_PO_4_, 0.05% Na_2_HPO_4 _·12 H_2_O, 40 ng/mL vitamin B_12_, and 80 ng/mL folic acid) containing 200 *μ*g/mL of streptomycin and 10 *μ*g/mL tetracycline. Transformants derived from Ax-2 cells were grown axenically by the shaking culture in PS-medium containing 200 *μ*g/mL of streptomycin, 10 *μ*g/mL of tetracycline, and 50 *μ*g/mL of G418. To allow cells to differentiate, cells were harvested during the exponential growth phase, washed once in BSS (Bonner's salt solution; 10 mM NaCl, 10 mM KCl, and 2 .7 mM CaCl_2_) [23] as starvation medium, and developed under the submerged conditions in BSS at 5 × 10^5^ cells/cm^2^. For the immunocytochemical observations, starved cells were settled on coverslips which were placed in glass dishes (3.6 cm diameter). This was followed by incubation at 22°C with or without 5 nM of TPA (Sigma). 

 Mouse C2C12 cells (myoblasts) were cultured at 37°C in 5% CO_2_ at 95% humidity in McCoy's 5A (GIBCO) supplemented with 10% of FBS (fetal bovine serum; Invitrogen, CA), 0.03% of L-glutamine, and 80 *μ*g/mL of kanamycin.

### 2.2. Vector Constructs

#### 2.2.1. The Vector Construct for Expression of *gfp/zyg1* Fusion Gene

The *v18-l-s65tgfp* (8181 bps) vector containing *v18* promotor and *s65tgfp* (green fluorescent protein with fast oxidizing mutation S65T) gene was used as the starting material. *Ubi *and* lac1* genes inserted between *v18* promotor and *s65tgfp* gene (984 bps) were deleted from this vector. To produce the *gfp/zyg1* fusion gene, the vector was treated by *Xho*I. *zyg1 *cDNA without initiation codon (ATG) was also treated by *Xho*I. A *Xho*I-treated *zyg1 *(-ATG) gene was inserted into a *Xho*I-treated *v18-l-s65tgfp* vector. Vectors in which *zyg1* gene was inserted in the downstream of *s65tgfp* gene at the sense direction were clonally selected (*gfp/zyg1* fusion gene).

#### 2.2.2. The Vector Construct for Expression of *ha/mzyg1* Fusion Gene

A humanized version (*mzyg1*) of *zyg1*cDNA (AB006956) was synthesized so that each amino acid codon was replaced by that most commonly found in mammalian cells (DNA 2.0 Inc). *mzyg1* treated by *Bam*HI and *Eco*RI was inserted into pUCD2 SR*α* [24] treated by *Bam*HI and *Eco*RI to create the *ha/mzyg1* fusion gene, in which the *mzyg1* gene was inserted in the downstream of a *ha *(3 × haemagglutinin) at the sense direction (*ha/mzyg1* fusion gene). The pUCD2 SR*α* vector was kindly gifted from Dr. K. Ohashi (Tohoku University).

#### 2.2.3. The Vector Construct for Expression of *gfp* and *mzyg1* Genes

For statistical analyses, a pIRES2-AcGFP vector (Clontech) was used. *mzyg1* treated by *Xba*I and *Eco*RI was inserted into the pIRES2-AcGFP vector treated by *Nhe*I and *Eco*RI to create a vector-expressing *gfp* and *mzyg1* genes (*gfp* and *mzyg1*).

### 2.3. Transformation of Ax-2 Cells

Ax-2 cells were grown by shake culture, harvested, and washed once with BSS. Starved cells were then washed twice with EB (electroporation buffer; 10 mM phosphate buffer (pH 6.2) containing 50 mM sucrose) and suspended in EB at 3 × 10^7^ cells/mL. Introduction of the vector constructs into Ax-2 cells was performed by electroporation, as described by Howard et al. [25], using 10 *μ*g of the vector containing *gfp* insert and 18 *μ*g of the vector containing *gfp/zyg1* insert, to gain the transformants GFP^CONT^ and GFP/ZYG1^OE^, respectively. The original transformant pool was first selected by incubation in PS medium containing 10 *μ*g/mL of G418 and finally cloned by selection in 50 *μ*g/mL of G418.

### 2.4. Western Blot Analysis

GFP^CONT^ and GFP/ZYG1^OE^ cells (1 × 10^7^) were boiled for 5 min in SDS-sample buffer (90 *μ*L) (2% SDS, 62.5 mM Tris-HCl, pH 6.8, 10% glycerol, 42 mM dithiothreitol, and 0.005% bromophenol blue) and then cooled on ice. The samples (3 *μ*L) were separated by 10% SDS-PAGE and transferred on Immunoblot PVDF membranes (Bio-Rad). The membranes were blocked overnight with TBS-T (20 mM Tris-HCl (pH 8.0), 150 mM NaCl, and 0.05% Tween 20) containing 0.5% BSA (bovine serum albumin, Sigma) at RT (room temperature). Subsequently, the membranes were probed by rabbit Phospho-(Ser) PKC Substrate antibody (Cell Signaling) diluted 1,000 times with enhancer A solution (Wako) for 1 h at RT. After two washings in TBS-T (10 min for each), the membranes were stained with goat HRP- (horseradish-peroxidase-) conjugated anti-rabbit secondary antibody (Amersham Biosciences) diluted 5,000 times with enhancer B solution (Wako) for 1 h. ImmunoStar (Long Detection, Wako) was used for the detection of HRP. After this detection, the membranes were washed in stripping solution (2-mercaptoethanol; 347 mL, 10% SDS; 10 mL, 0.5 M Tris-HCl (pH 6.7); 6.25 mL, DW; 33.4 mL) for 30 min at 50°C, blocked 1 h with TBS-T containing 5% skim milk, and then probed by the rabbit anti-GFP antibody (Invitrogen) diluted 500 times with TBS-T containing 5% skim milk for 1 h at RT. After two washings in TBS-T (10 min for each), the membranes were stained for 1 h with the goat HRP-conjugated anti-rabbit secondary antibody (Amersham Biosciences) diluted 5,000 times with TBS-T. ImmunoStar (Long Detection, Wako) was used for the detection of HRP.

### 2.5. Immunocytochemical Staining of GFP^CONT^ and GFP/ZYG1^OE^ Cells

Growing GFP^CONT^ and GFP/ZYG1^OE^ cells were harvested, washed once with BSS, and resuspended in BSS at 2.5 × 10^6^ cells/mL. Two mL of the cell suspension was dropped on coverslips placed in glass dishes (diameter; 3.6 cm) and incubated for 2 h. The cells adhering to the coverslips were fixed with 4% paraformaldehyde for 20 min or ice-cold methanol for 10 min. In the case of fixation by paraformaldehyde, it was followed by treatment with 0.2% Triton X-100 in PBS containing 2% FBS for 30 min at RT to obtain permeability. Then, the samples were soaked in PBS (10 mM phosphate Na_2_/K buffer, pH 7.0, 0.9% NaCl), blocked with PBS containing 2% FBS (fetal bovine serum) for 30 min at RT, and probed with the rabbit Phospho-(Ser) PKC Substrate antibody (Cell Signaling) diluted 300 times with PBS containing 2% FBS, for 1 h at RT. The samples were washed by three changes of PBS and then stained with the goat rhodamine-conjugated secondary anti-rabbit IgG (H+L) (Thermo) for 1 h at RT. After washing in PBS, samples were mounted in PBS containing 20% glycerol.

### 2.6. Introduction of Vectors into C2C12 Cells (Myoblasts)

C2C12 cells (myoblasts) were placed on coverslips and transfected with the vector containing a *ha *gene, a *ha/mzyg1 *fusion gene, a *gfp *gene, or *gfp* and *mzyg1* genes using Lipofectamine 2000 (Invitrogen) according to the manufacturer's instruction. After 6 h of transfection in Opti-MEM (Invitrogen) without FBS, samples were washed twice with PBS and incubated with DMEM (Dulbecco's Modified Eagle Medium, Invitrogen) containing 10% FBS and 80 *μ*g/mL of kanamycin for 24 h at 37°C in 5% CO_2_ at 95% humidity.

### 2.7. Immunocytochemical Staining of C2C12 Cells Expressing HA or HA/ZYG1 Protein

After transfection, the samples were fixed with PBS containing 4% paraformaldehyde for 20 min and permeabilized with 0.2% Triton X-100 in PBS containing 2% FBS for 30 min at RT, followed by blocking with PBS containing 2% FBS for 30 min at RT. After three washings in PBS, the samples were stained with the mouse anti-HA antibody (Roche Applied Science) diluted 400 times with PBS containing 2% FBS, for 1 h at RT. Then they were washed with three changes of PBS and stained with the goat FITC-conjugated secondary anti-mouse IgG (H+L) (Alexa Fluor 488, Invitrogen) in PBS containing 2% FBS for 1 h at RT. After washings in PBS, the samples were mounted in PBS containing 20% glycerol and DAPI (4′-6-diamidino-2-phenylindole) (final concentration: 2.5 *μ*g/mL) (Wako, Japan).

### 2.8. Photographs

Photographs were taken under fluorescence microscope (Axioskop, Zeiss) or confocal microscope (Fluoview FV 1000, Olympus).

## 3. Results

### 3.1. Localization of GFP/ZYG1 Fusion Protein

From our previous studies [10, 20], ZYG1 was expected to translocate to the cell membrane where cell fusion occurs and probably coupled with its phosphorylation by PKC. To examine this possibility, the *gfp/zyg1* fusion gene was introduced into *D. discoideum* Ax-2 cells. In the present work, we used Ax-2 cells, instead of *D. mucoroides 7* (Dm7) that had been used in our previous works, because Ax-2 cells with 10-11 *μ*m of diameter are bigger than Dm7 cells with 6-7 *μ*m of diameter and more convenient for cytological studies. In addition, it is suggested that the mechanism of zygote formation might be fundamentally common [10, 12]. The vector containing only a *gfp *gene was also introduced into Ax-2 cells as a control. Cells overexpressing GFP/ZYG1 (GFP/ZYG1^OE^) and overexpressing GFP (GFP^CONT^) were selected by 50 *μ*g/mL of G418, and the localization of GFP/ZYG1 fusion protein or GFP protein was monitored under fluorescence and confocal microscopes. 

**Figure 1:**
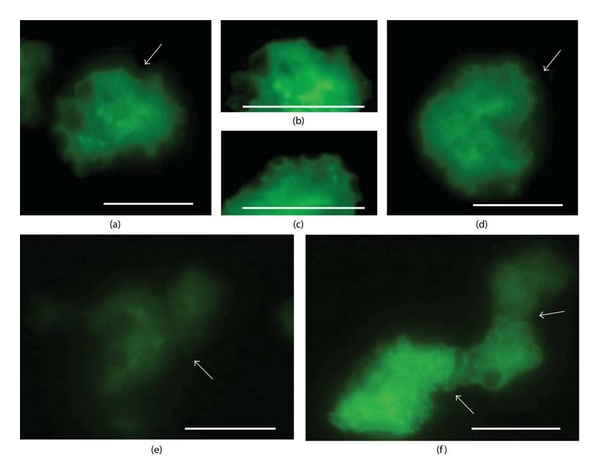
Localization of GFP and GFP/ZYG1 fusion protein. GFP^CONT^ and GFP/ZYG1^OE^ cells are starved for 2 h, fixed with 4% paraformaldehyde for 20 min, and observed under a fluorescence microscope. GFP protein in GFP^CONT^ cells and GFP/ZYG1 fusion protein in GFP/ZYG1^OE^ cells are shown as the green color of GFP. GFP/ZYG1 fusion protein is preferentially localized at the periphery of vesicles (c, d; arrow) and at the phagocytic cups which are attached to form cell-cell contacts (f; arrows). On the other hand, in GFP^CONT^ cells, GFP protein is uniformly distributed in the cytoplasm (a, b; arrow), and its localization at the region of cell-cell contacts is not observed (e; arrow). This indicates that its localization is due to the presence of ZYG1, but not to GFP itself. Photographs of (b) and (c) are the magnified (a) and (d), respectively, and adjusted to get brighter images. Bars: 20 *μ*m.

GFP protein in GFP^CONT^ cells and GFP/ZYG1 fusion protein in GFP/ZYG1^OE^ cells were observed as the green color of GFP. When GFP^CONT^ cells were developed for 2 h after starvation, GFP protein was uniformly distributed in the cytoplasm (Figures 1(a) and 1(b)). On the other hand, in GFP/ZYG1^OE^ cells, most GFP/ZYG1 fusion protein was localized at the periphery of vesicles (Figures 1(c) and 1(d); arrow), particularly around the phagocytic cups. The phagocytic cups became larger and attached to each other when cell-cell contacts were formed (Figure 1(f); arrows). Since the localization of GFP at the periphery of vesicles and the region of cell-cell contacts was never observed in GFP^CONT^ cells (Figures 1(a) and 1(e); arrows), it is most likely that its localization is due to the presence of ZYG1, but not to GFP itself. Successive observation of intact cells observed under a fluorescence microscope has showed that GFP/ZYG1 fusion protein translocates to the cell cortex and then detaches from the cell cortex within 10 sec (Figure S1 see in supplementary material available online at doi:10.1155/2012/657423). Thus, the ZYG1 localization seemed to change temporarily.

**Figure 2:**
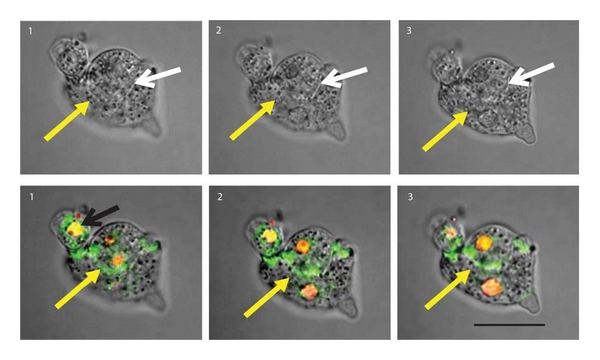
Behavior of ZYG1 during cell fusion. GFP/ZYG1^OE^ cells were starved for 2 h and fixed with methanol for 10 min. Pictures of their serial sections (1~3 : 4 *μ*m thick) were taken under a confocal microscope. Pictures on the upper side show DIC images and those on the lower side the merged images of DIC, GFP, and DAPI staining. The color of DAPI staining is shown as red. In the DIC images (upper panels), the boundaries shown by white arrows are clear in all sections. On the other hand, the boundary shown by a yellow arrow is clearly observed in section  1, while the demarcation becomes unclear in sections  2 and 3. This indicates that the two cells are fusing around here (2, 3). In merged images (lower panels), GFP/ZYG1 fusion protein is localized at the region of cell fusion (2, 3; yellow arrows). Nuclei become yellow by merging GFP with DAPI staining. Bar: 20 *μ*m.

 When optical sections of GFP/ZYG1^OE^ cells under a confocal microscope were observed, a part of two contacted cells seemed to fuse to each other because demarcation was lost in sections  2 and  3 of Figure 2 (DIC; yellow arrows). Considering the merged images of DIC (Differential Interference Contrast) and green fluorescence emitted from GFP, it is quite possible that GFP/ZYG1 fusion protein is preferentially localized at the region of cell fusion (Figure 2). It was also observed that GFP protein was localized in nuclei (shown as red in Figure 2). These results showed that ZYG1 was predominantly localized at the regions of cell-cell contacts and cell fusion.

### 3.2. Phosphorylation of ZYG1 by PKC

**Figure 3:**
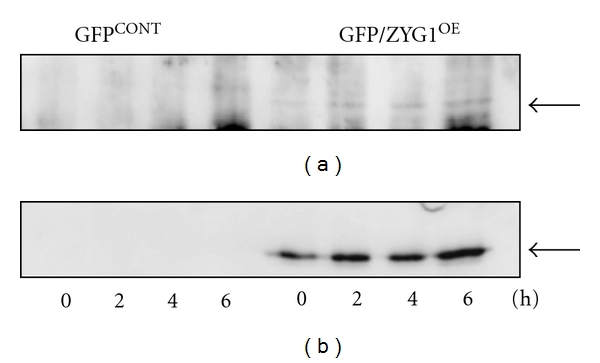
Phosphorylation of GFP/ZYG1 fusion protein by cPKC. GFP^CONT^ and GFP/ZYG1^OE^ cells were collected at 2-hour intervals after starvation. Each sample was separated by 10% SDS-PAGE, followed by Western blottings using the rabbit Phospho-(Ser) PKC Substrate antibody and the rabbit anti-GFP antibody. Hours after starvation are designated at the bottom. The GFP/ZYG1 fusion protein (67.4 kDa) is phosphorylated by cPKC (a; arrow), while no bands around 67.4 KD are detected in the samples obtained from GFP^CONT^ cells. GFP/ZYG1^OE^ cells also express GFP protein, as detected by the anti-GFP antibody (b; arrow).

To know if ZYG1 is phosphorylated by PKC (protein kinase C), ZYG1 phosphorylation in GFP/ZYG1^OE^ cells was examined by Western blotting using the antibody that specifically recognized the serine residues phosphorylated by PKC. Results showed that a small amount of GFP/ZYG1 fusion protein (67.4 kDa) was phosphorylated by PKC during development (Figure 3(a)). The same bands were also recognized by the detection of GFP using the anti-GFP antibody (Figure 3(b)). In GFP^CONT^ cells, the band of GFP protein was detected at 38 kDa (Figure S2 see in supplementary material available online at doi:10.1155/2012/657423). Since the antibody (Phospho-(Ser) PKC Substrate antibody) used in this study reacts specifically to the phosphoserine substrate phosphorylated by cPKC (conventional PKC which requires Ca^2+^ and DAG (diacylglycerol) for maximal activity), it is clear that ZYG1 protein is actually phosphorylated by cPKC. 

**Figure 4:**
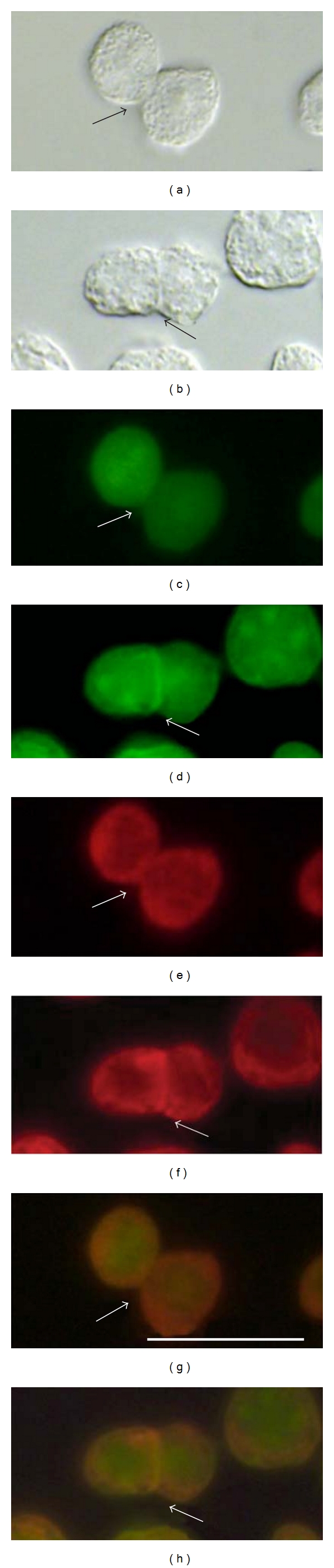
Immunocytochemical detection of the PKC-mediated ZYG1 phosphorylation. Starved GFP^CONT^ and GFP/ZYG1^OE^ cells were developed for 2 h, fixed with methanol, and stained by the Phospho-(Ser) PKC Substrate antibody. This was followed by the rhodamine-conjugated anti-rabbit secondary antibody to detect the proteins phosphorylated by cPKC, as described in Section 2. Photographs were taken under DIC (a and b) and fluorescence microscope (GFP, (c) and (d); Rhodamine, (e) and (f); GFP and Rhodamine merged, (g) and (h)). Photographs represent the same fields of GFP^CONT^ cells (a, c, e, g) and of GFP/ZYG1^OE^ cells (b, d, f, h). GFP protein in GFP^CONT^ cells and GFP/ZYG1 fusion protein in GFP/ZYG1^OE^ cells are shown as the green color of GFP (c and d). In GFP/ZYG1^OE^ cells, GFP/ZYG1 fusion protein is localized at the region of cell-cell contacts (d; arrow). On the other hand, the localization of GFP protein at the region of cell-cell contacts is not observed in GFP^CONT^ cells (c; arrow). This indicates that its localization is due to the presence of ZYG1, but not to GFP itself. The protein phosphorylated by PKC is shown as the red color of Rhodamine in both cells ((e) and (f)). The protein phosphorylated by PKC is also localized at the region of cell-cell contacts in GFP/ZYG1^OE^ cells (f; arrow). This localization shown by Rhodamine is not observed in GFP^CONT^ cells (e; arrow). Since the merged color of GFP and Rhodamine shows yellow in GFP/ZYG1^OE^ cells (h; arrow), it is evident that ZYG1 protein is phosphorylated by cPKC at the regions of cell-cell contacts. Bar: 25 *μ*m.

The phosphorylation of ZYG1 protein was examined also by immunocytochemical staining. As TPA, a potent activator of cPKC, is known to enhance the formation of zygotes [9], experiments were carried out with or without TPA (5 nM). In GFP/ZYG1^OE^ cells, GFP/ZYG1 fusion protein shown as the green color of GFP was localized at the region of cell-cell contacts (Figure 4(d); arrow). On the other hand, the localization of GFP protein at the region of cell-cell contacts was not observed in GFP^CONT^ cells (Figure 4(c); arrow). This indicates that ZYG1 protein, but not GFP, localizes at the region of cell-cell contacts in GFP/ZYG1^OE^ cells, as shown already in Figure 1. The protein phosphorylated by PKC shown as the red color of Rhodamine was also localized at the region of cell-cell contacts in GFP/ZYG1^OE^ cells (Figure 4(f); arrow). This localization was not observed in GFP^CONT^ cells (Figure 4(e); arrow). When GFP was merged with Rhodamine, the image exhibited yellow color in GFP/ZYG1^OE^ cells (Figure 4(h); arrow). This means that ZYG1 protein was colocalized with the proteins phosphorylated by cPKC at the regions of cell-cell contacts (Figure 4, arrows). Naturally enough, the colocalization of GFP and Rhodamine was not observed in GFP^CONT^ cells (Figure 4(g); arrow). 

**Figure 5:**
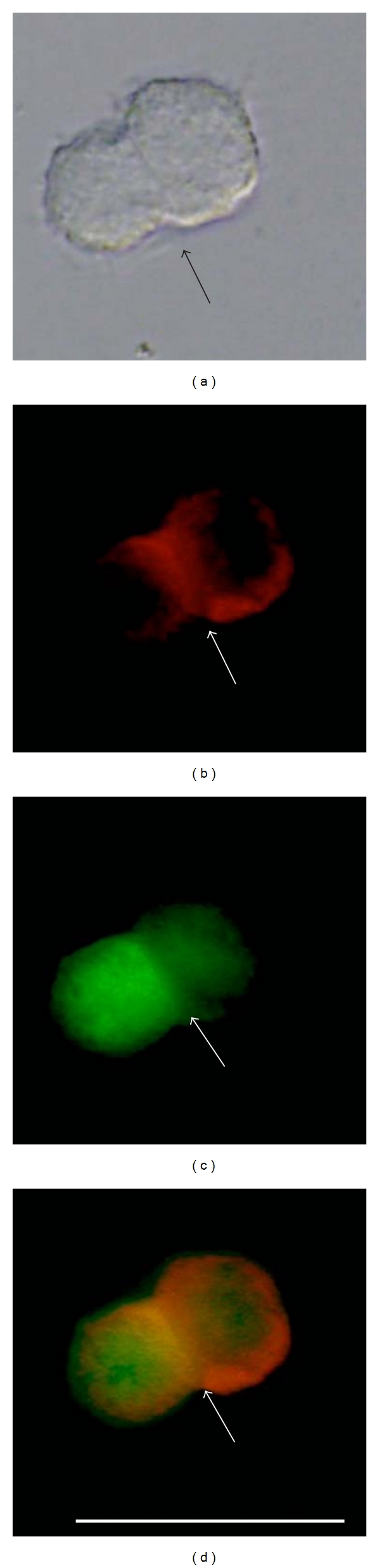
The phosphorylation of GFP/ZYG1 fusion protein by cPKC occurs at the region of cell fusion. Starved GFP/ZYG1^OE^ cells were developed for 2 h in the presence of TPA (5 nM). The sample of GFP/ZYG1^OE^ cells for the immunocytochemical detection was prepared as described in Figure 4. The same fields are shown as images of DIC (a), Rhodamine (b), GFP (c), and GFP and Rhodamine merged (d). Since it is evident by the DIC image that there is no boundary between the two cells (a; black arrow), cell fusion is occurring between two cells. Since the merged image shows yellow color (d; a white arrow), it is evident that GFP/ZYG1 fusion protein and proteins phosphorylated by cPKC are colocalized particularly at the region where cell fusion occurred. Bar: 25 *μ*m.

Multinucleate cells were formed more frequently in GFP/ZYG1^OE^ cells that had been incubated with TPA, as compared with those incubated without TPA, thus suggesting that cell fusion in GFP/ZYG1^OE^ cells might be accelerated by TPA. Since there is no boundary between the two cells in GFP/ZYG1^OE^ cells incubated with TPA (Figure 5(a); black arrow), it is quite likely that cell fusion actually occurs between them. GFP/ZYG1 fusion protein was co-localized with the proteins phosphorylated by cPKC at the region of cell fusion, because the image exhibited yellow color around the region of cell fusion occurred (Figure 5; arrows). These results suggest that ZYG1 is phosphorylated by PKC at the region of cell-cell contacts and cell fusion. 

Interestingly, the region where cell-cell contacts and cell fusion occurred was found to be composed of a cluster of small vesicles (Figure 2). 

### 3.3. Enforced Expression of ZYG1 Protein in Mouse Myoblasts Induces Their Fusion

**Figure 6:**
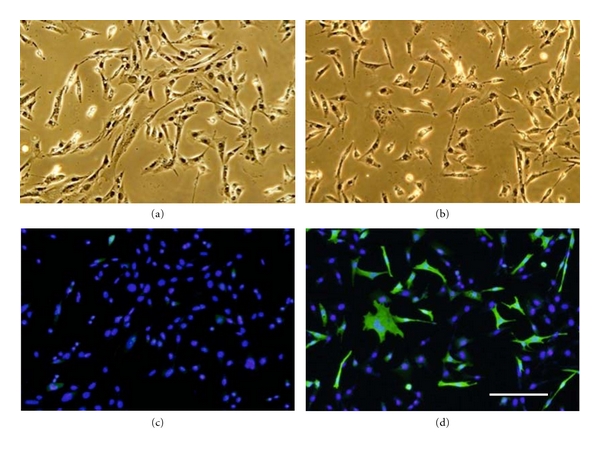
Behavior of C2C12 cells (myoblasts) transfected with the* ha/mzyg1* fusion gene. C2C12 cells were transfected with the *ha* gene (control; a, c) or *ha/mzyg1 *fusion gene (HA/ZYG1; b, d), fixed with 4% paraformaldehyde and stained by antibodies and DAPI according to the methods described in Section 2. Phase-contrast micrographs on the upper sides represent the same field as the merged images of FITC and DAPI on the lower sides ((a) and (c), (b) and (d)). C2C12 cells expressing the HA/ZYG1 fusion protein stained with the anti-HA antibody and then FITC-conjugated anti-mouse secondary antibody are detected as the green-colored cells under a fluorescence microscope though a considerable number of cells are intermingled as untransfected ones in the population (d). Nuclei stained with DAPI are shown as purple color by UV-excitation (c and d). The transfection rate with the *ha/mzyg1* gene was 36.8 ± 8%. The expression of HA protein in control cells was scarcely detected (c). Bar: 100 *μ*m.

Ca^2+^ and PKC are known to be involved in myoblast fusion during myogenesis [15–17] and in zygotic cell fusion of *Dictyostelium *cells [6–9]. Therefore, it is possible that there may be a common signal transduction pathway for cell fusion in both of the cell lines. To know if ZYG1 is able to induce myoblasts, a *ha/zyg1* fusion gene was transfected into C2C12 cells (myoblasts). In spite of many trials, however, ZYG1 protein was never expressed in C2C12 cells. For the purpose of ZYG1 expression in mammalian cells, a humanized version (*mzyg1*) of the *zyg1* gene was synthesized in which each amino acid codon was replaced by that most commonly found in mammalian cells (DNA 2.0 Inc.). When a *ha/mzyg1* fusion gene (*ha/mzyg1*) was transfected into C2C12 cells, the expression of HA/ZYG1 fusion protein was revealed by the anti-HA antibody (mouse) and then the FITC-conjugated secondary anti-mouse antibody. As a control, C2C12 cells transfected with a vector containing only a *ha* gene were prepared. At 24 h of incubation in DMEM containing 10% FBS after transfection, cells were fixed with 4% paraformaldehyde, followed by immunostaining. As a result, cells expressing HA/ZYG1 fusion protein (HA/ZYG1) were recognized because of their green color of fluorescence (Figure 6(d)). The transfection rate was 36 ± 8 %. The expression of HA protein in control cells was scarcely detected though the HA gene was present in the vector used for transfection (Figure 6(c)). 

**Figure 7:**
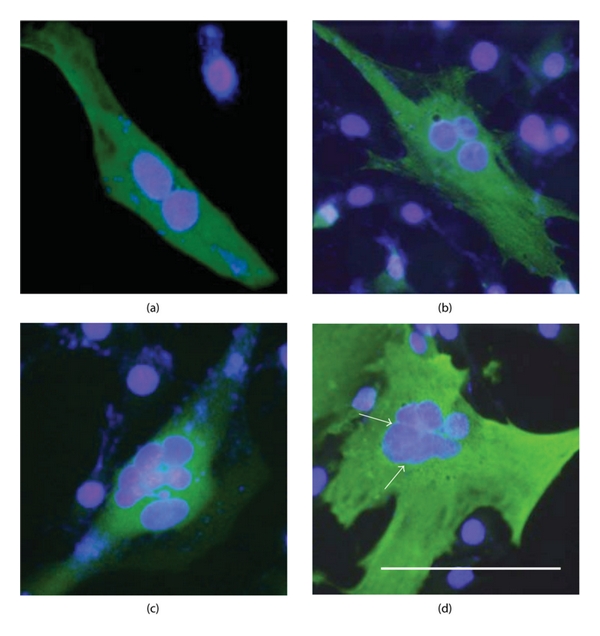
Formation of multinucleate cells in C2C12 cells transfected with the *ha/mzyg1* fusion gene (HA/ZYG1). C2C12 cells were transfected with the *ha/mzyg1* fusion gene, fixed with 4% paraformaldehyde, and stained by antibodies and DAPI according to the methods described in Section 2. The FITC images merged with DAPI images of large-sized HA/ZYG1 cells containing 2 nuclei (a), 3 nuclei (b), and 7 nuclei (c) are shown. Considering some gulfs observed in a nucleus, it is evident that a large nucleus is formed by nuclear fusion (d; arrows). Bar: 100 *μ*m.

 Some HA/ZYG1 cells showed a typical spindle shape as a sign of myoblast fusion (Figure S3(A) see in supplementary material available online at doi:10.1155/2012/657423) though a large number of cells exhibited fibroblast-like morphology. The spindle-shaped cells showed a characteristic alignment that was the parallel apposition of the long axes of myoblasts forming end-to-end alignment as a sign of acquisition of fusion competence (Figure S3(B) see in supplementary material available online at doi:10.1155/2012/657423) [18]. In addition, HA/ZYG1 cells formed giant multinucleate cells frequently (Figure 7) and exhibited fibroblast-like morphology. As binucleate cells were frequently observed to have larger sizes of cells and nuclei (Figure 7(a)), it is quite different from the cells at the mitosis (Figure S4 see in supplementary material available online at doi:10.1155/2012/657423). The highly elongated cells known as a sign of typical myoblast fusion, in which nuclei were arranged in a single file, were not observed. In addition, nuclei tended to fuse and form a large size of a fused nucleus or nuclei (Figure7(d)) though this event was never observed in the normal process of myoblast fusion.

**Figure 8:**
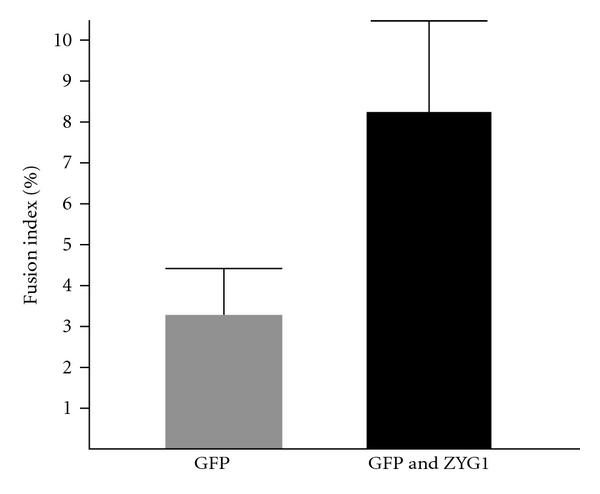
Enhanced formation of multinucleate cells by transfecting with the *mzyg1 *gene. The percentages of cells containing more than two nuclei are shown as fusion indexes. The pIRES2-AcGFP vector or pIRES2-AcGFP vector containing *mzyg1 *gene was transfected into C2C12 cells as described in Section 2. After 1 day, cells were fixed with 4% paraformaldehyde and mounted in PBS containing 20% glycerol and DAPI (final concentration: 2.5 *μ*g/mL) after three washings with PBS. The numbers of cells and nuclei stained with DAPI were counted. Three samples in two independent experiments were counted (about 500*∼*1,000 cells/sample). The fusion index (ratio of the cell number participated in cell fusion to the total cell number) was calculated by the following formula: Fusion index (%) = 2 × (The number of cells containing 2 nuclei) + 3 × (The number of cells containing 3 nuclei)⋯*n* × (the number of cells containing *n* nuclei)/1 × (The number of cells containing 1 nucleus) + 2 × (The number of cells containing 2 nuclei) + 3 × (the number of cells containing 3 nuclei⋯*n* × (The number of cells containing *n* nuclei) × 100. GFP; C2C12 cells transfected with the pIRES2-AcGFP vector. GFP and ZYG1; C2C12 cells transfected with the pIRES2-AcGFP vector containing the *mzyg1* gene. Bars: standard deviations.

 Since the characteristics described above were also observed in control cells, the numbers of cells having multinuclei were counted and statistically analyzed using a pIRES2-AcGFP vector, which contains the *gfp *gene and expresses constitutively GFP protein independently from the gene inserted. The expression of GFP was weak, but significantly higher compared to GFP-uninfected cells (Figure S5 see in supplementary material available online at doi:10.1155/2012/657423). By transfection with the pIRES2-AcGFP vector or pIRES2-AcGFP vector containing an *mzyg1* gene, almost all of the cells expressed GFP or GFP and ZYG1 protein, respectively (Figure S6 see in supplementary material available online at doi:10.1155/2012/657423). Thus, the rates of cell fusion, which were designated as fusion indexes, were found to be significantly higher in cells transfected with the vector containing an *mzyg1* gene than the control cells (Figure 8). The difference of fusion index between GFP cells and GFP and ZYG1 cells was statistically significant (*P* < 0.002), indicating that the myoblast fusion is markedly enhanced by the *mzyg1 *gene. These results indicate that ZYG1 is able to cause cell fusion in myoblasts.

## 4. Discussion

Previous studies have suggested that the signal transduction pathway via Ca^2+^ and PKC may participate in sexual fusion (zygote formation) of *Dictyostelium *cells [10, 12]. Since ZYG1 protein has several deduced sites phosphorylated by PKC, ZYG1 protein was expected to be a likely substrate of PKC. Provided that ZYG1 is phosphorylated by PKC prior to cell fusion, ZYG1 protein will be translocated to the cell membrane where cell fusion occurs.

As presented in this study, the ZYG1 protein was found to actually translocate to the cell cortex including the cell membrane, where cell-cell contacts and subsequent cell fusion occur. Importantly, ZYG1 localizes at the periphery of vesicles including phagocytic cups. Since cell-cell contacts are often observed between phagocytic cups of two facing cells, it is likely that cell fusion may occur through the fusion of those phagocytic cups. The phosphorylation by cPKC of ZYG1 at the regions of cell-cell contacts and cell fusion was verified by Western blot analysis and immunocytochemical study. PKC is a multigene family of serine/threonine (ser/thr) kinases that play critical roles in many signal transduction pathways [26, 27]. This kinase family is composed of at least 11 different isozymes classified into the following 3 groups: (1) cPKC (conventional) *α*, *β*, and *γ* which require Ca^2+^ and DAG (diacylglycerol) for maximal activity [28]; (2) nPKC (novel) *δ*, *ε*, *η*, *μ*, and *θ* isoforms, which are Ca^2+^ independent but require DAG [29–32]; (3) aPKC (atypical) *ζ*, *λ*, and *τ*, which are Ca^2+^ and DAG insensitive [33, 34]. Since Ca^2+^ is required for sexual fusion in *Dictyostelium *cells [2–4], we used the Phospho-(Ser) PKC Substrate antibody that specifically recognizes cPKC substrates containing phosphoserine. As a result, we found that ZYG1 protein was phosphorylated by cPKC among the PKC family. After homology search, ZYG1 protein has seven sites phosphorylated by cPKC [20]. Among the seven deduced phosphorylation sites, five are serine residues though it is presently unknown if some or all of the serine residues are phosphorylated by cPKC for cell fusion. There are two threonine-phosphorylation sites, but their phosphorylation by cPKC was not detected in our preliminary experiment.

 Considering the data presented here, a possible signal transduction pathway involved in sexual cell fusion has been proposed, as follows: (1) cPKC is activated by the increase of intracellular Ca^2+^. (2) ZYG1 is translocated to the cell membrane. (3) ZYG1 protein is phosphorylated by activated cPKC. (4) The sexual cell fusion is induced by the phosphorylated ZYG1 protein.

 Since cPKC is known to be involved in myoblast fusion [15–17], the fusion processes in myoblasts and *Dictyostelium *cells might share a similar signal transduction pathway. When C2C12 cells (myoblasts) were transfected with the fusion gene of *ha *and humanized *zyg1* (*ha/mzyg1*) or pIRES2-AcGFP vector containing the *mzyg1* gene (*gfp and mzyg1*), the process of myoblast fusion was found to be accelerated. Since ZYG1 has not a mammalian ortholog, protein(s) other than ZYG1 may be phosphorylated by PKC during myogenesis. However, the result presented here has suggested that ZYG1 is able to take place of the protein phosphorylated by PKC in mammalian cells. Also, the protein phosphorylated by PKC during myoblast fusion may be functionally similar to ZYG1, though the target protein of PKC in myoblasts remains to be identified. The multinucleate cells formed by the transfection with *mzyg1*, however, exhibited fibroblast-like morphology, while highly elongated cells in which nuclei arrange in a single file as observed in the normal process of myoblast fusion were not formed. The following two explanations for this difference might be possible. During the transfection carried out without FBS for 6 h, myoblasts stop their growth and start differentiation. After a short period of differentation, myoblasts restart growth in the medium containing FBS. As a result, the multinucleate cells formed by cell fusion assume fibroblast-like morphology. Another possibility is that HA/ZYG1 cells might be able to fuse by ZYG1 even under the growth conditions. In all events, it is of importance to note that a novel protein, ZYG1, has an inductive effect on myoblast fusion as well as on sexual fusion of *Dictyostelium* cells.

## Supplementary Material

Figure S1: Behavior of GFP/ZYG1 protein in intact GFP/ZYG1OE cells.
Figure S2: Detection of GFP protein and GFP/ZYG1 fusion protein by the anti-GFP antibody.
Figure S3: Acquisition of the fusion competence in myoblasts transfecting *ha/mzyg1* fusion gene.
Figure S4: Mitotic division observed in C2C12 cells.
Figure S5: Comparison of intact C2C12 cells with those transfected with the pIRES-AcGFP vector.
Figure S6: GFP images merged with DIC images in C2C12 cells transfected with the pIRES2-AcGFP vector (A) and in C2C12 cells transfected with the pIRES2-AcGFP vector containing the *mzyg1* gene (B).
