# Supplementary material for: PKC-Mediated ZYG1 Phosphorylation Induces Fusion of Myoblasts as well as of Dictyostelium Cells
Source: Int J Cell Biol. 2012 Feb 6;2012:657423. doi: 10.1155/2012/657423 (PMC3296296; doi:10.1155/2012/657423)
Supplement: Supplementary file 1 — Figure S1: Behavior of GFP/ZYG1 protein in intact GFP/ZYG1OE cells. Figure S2: Detection of GFP protein and GFP/ZYG1 fusion protein by the anti-GFP antibody. Figure S3: Acquisition of the fusion competence in myoblasts transfecting ha/mzyg1 fusion gene. Figure S4: Mitotic division observed in C2C12 cells. Figure S5: Comparison of intact C2C12 cells with those transfected with the pIRES-AcGFP vector. Figure S6: GFP images merged with DIC images in C2C12 cells transfected with the pIRES2-AcGFP vector (A) and in C2C12 cells transfected with the pIRES2-AcGFP vector containing the mzyg1 gene (B). [file 657423.f1.pdf]

Supplemental figures and their legends

S1.

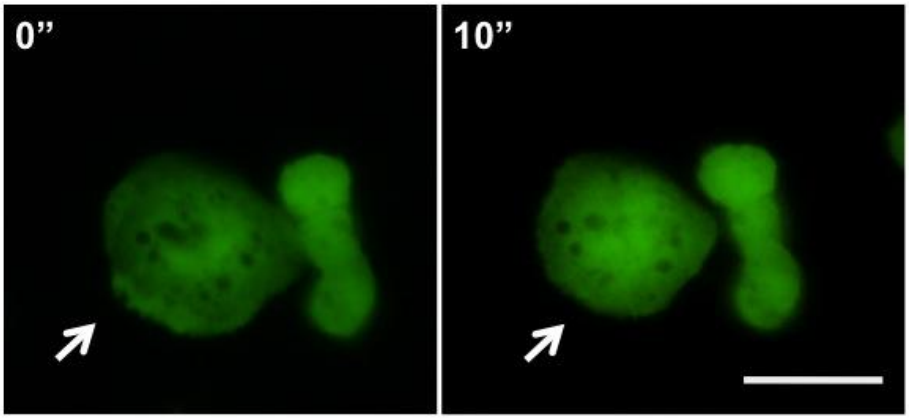

Behavior of GFP/ZYG1 protein in intact GFP/ZYG1^{OE} cells. Starved cells were settled on coverslips which were placed in glass dishes (3.6 cm diameter) and incubated for 4 h. Cells attached on the coverslips were observed under a fluorescence microscope. GFP/ZYG1 protein observed on the cell cortex of a large cell (0”) becomes non-detectable after 10 sec (10”) (white arrows). Bar, 20 μm.

S2.

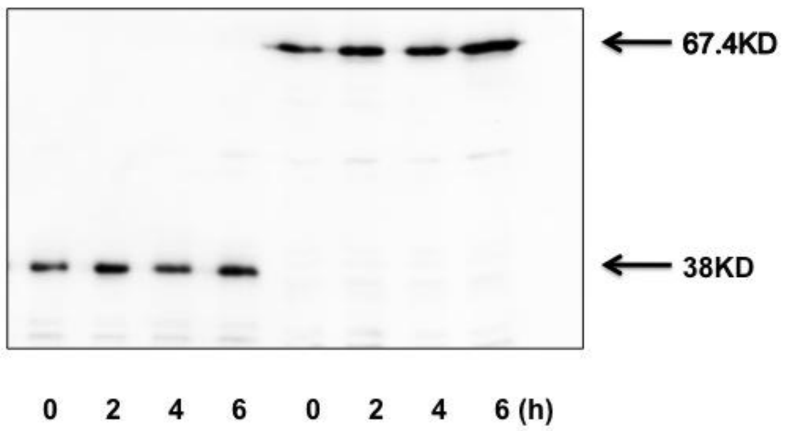

Detection of GFP protein and GFP/ZYG1 fusion protein by the anti-GFP antibody. GFP^{CONT} and $GFP/ZYG1^{OE}$ cells were separately harvested at the growth phase and loading samples were prepared according to the methods as described in Materials and methods. Each sample was separated by 10 % SDS-PAGE, followed by staining with the anti-GFP antibody. The bands of GFP are detected at 38 kDa for GFP protein and 67.4 kDa for GFP/ZYG1 fusion protein, as shown by arrows. Developmental times are indicated at the bottom of the figure.

S3.

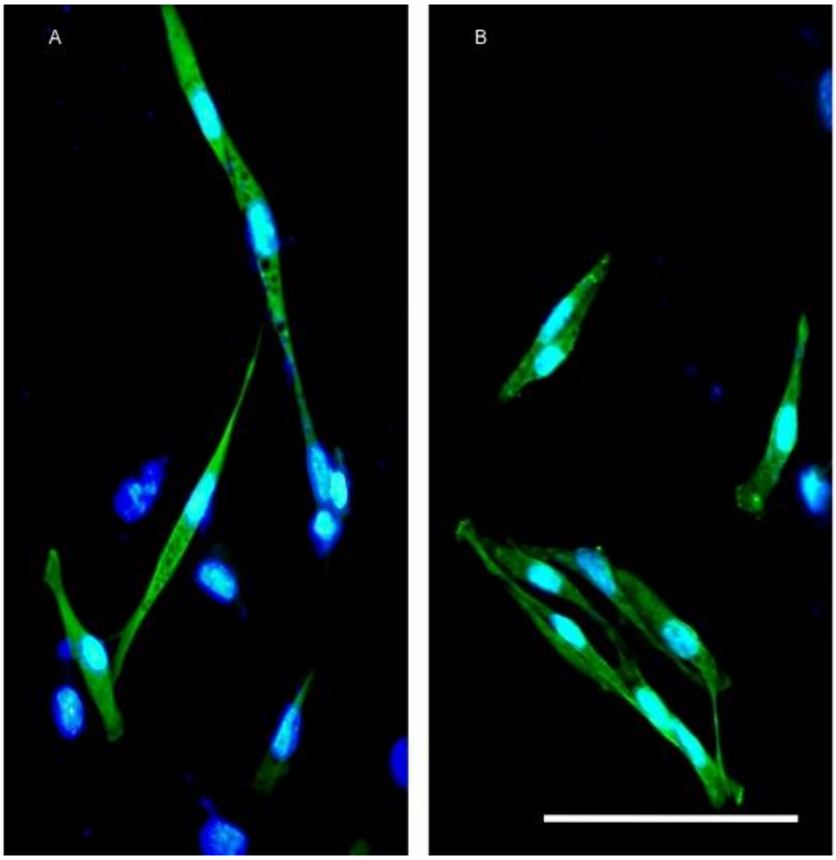

Acquisition of the fusion competence in myoblasts transfecting *ha/mzyg1* fusion gene. C2C12 cells (mouse myoblasts) were transfected with *ha/mzyg1* fusion gene, fixed with 4% paraformaldehyde and stained by the FITC-conjugated anti-HA antibody and DAPI according to the methods described in Materials and methods. The FITC images merged with DAPI images in HA/ZYG1 cells show the typical spindle-shaped cells (A), the formation of parallel alignment (B) and end-to-end alignment (A and B) as a sign of the acquisition of fusion competence. Bar, 100 μm,

S4.

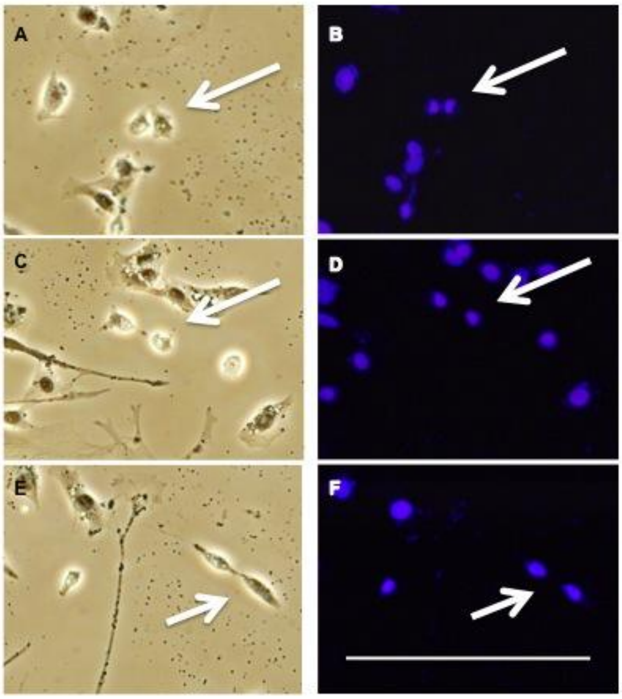

Mitotic division observed in C2C12 cells. Photographs on the right sides represent DAPI-stained images (B, D, F) in the same fields as the phase-contrast micrographs on the left side (A, C, E). Dividing cells seem to be smaller in size and have smaller nuclei (arrows). Bar, 100 μm.

S5.

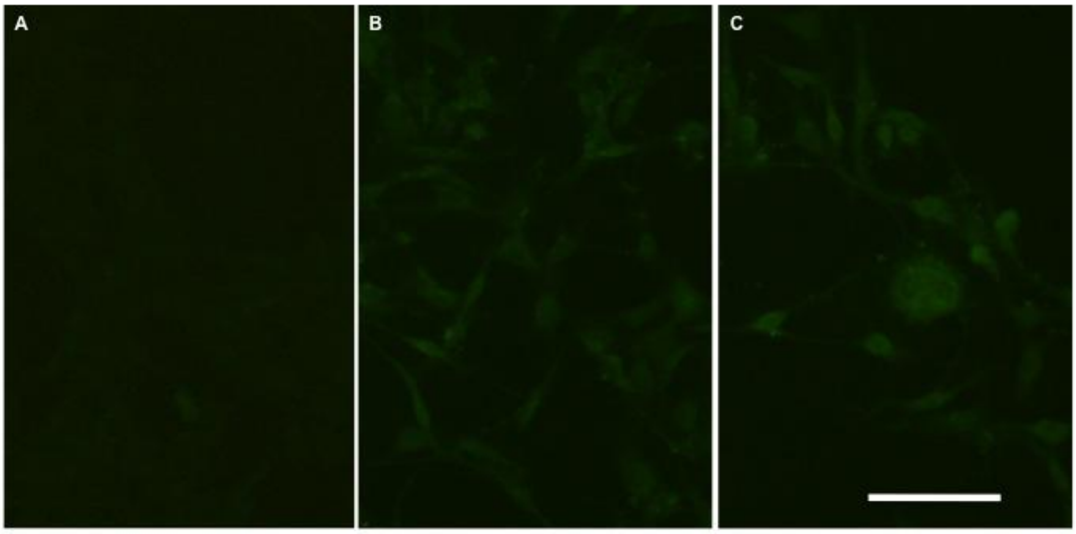

Comparison of intact C2C12 cells with those transfected with the pIRES-AcGFP vector. (A) C2C12 cells, (B) C2C12 cells transfected with the pIRES-AcGFP vector, and (C) C2C12 cells transfected with the pIRES-AcGFP vector containing *mzyg1* gene. C2C12 cells transfected with the pIRES-AcGFP vector with or without *mzyg1* gene express weak GFP fluorescence. However, it is evident that the fluorescence of GFP is distinct from that in non-treated C2C12 cells. Bar, 100 μm.

S6.

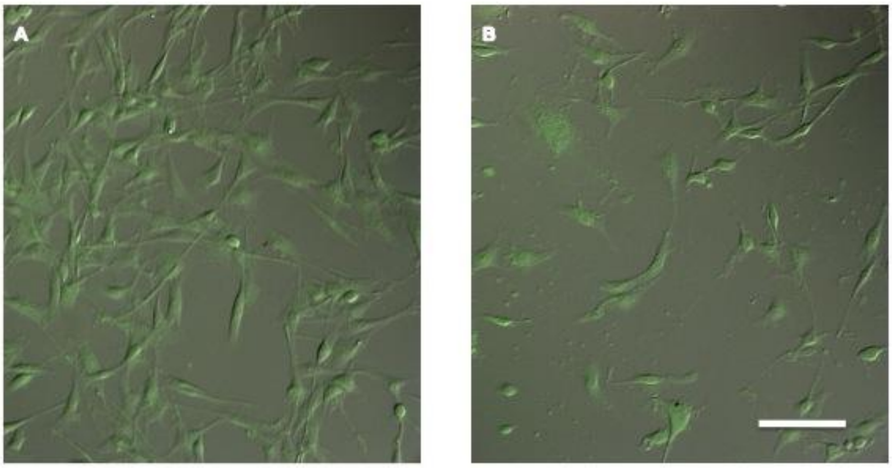

GFP images merged with DIC images in C2C12 cells transfected with the pIRES2-AcGFP vector (A) and in C2C12 cells transfected with the pIRES2-AcGFP vector containing the *mzyg1* gene (B). It is evident that GFP protein is expressed in almost all of the cells transfected with the pIRES2-AcGFP vector or pIRES2-AcGFP vector containing the *mzyg1* gene. Bar, 100 µm.
